# Effect of Precursor Stoichiometry on the Performance and Stability of MAPbBr_3_ Photovoltaic Devices

**DOI:** 10.1002/ente.201900737

**Published:** 2019-08-20

**Authors:** Lukas M. Falk, Katelyn P. Goetz, Vincent Lami, Qingzhi An, Paul Fassl, Jonas Herkel, Fabian Thome, Alexander D. Taylor, Fabian Paulus, Yana Vaynzof

**Affiliations:** ^1^ Kirchhoff Institute for Physics University of Heidelberg Im Neuenheimer Feld 227 69120 Heidelberg Germany; ^2^ Centre for Advanced Materials University of Heidelberg Im Neuenheimer Feld 225 69120 Heidelberg Germany

**Keywords:** lead bromide perovskites, photovoltaic devices, reproducibility, stability, stoichiometry

## Abstract

The wide‐bandgap methylammonium lead bromide perovskite is promising for applications in tandem solar cells and light‐emitting diodes. Despite its utility, there is a limited understanding of its reproducibility and stability. Herein, the dependence of the properties, performance, and shelf storage of thin films and devices on minute changes to the precursor solution stoichiometry is examined in detail. Although photovoltaic cells based on these solution changes exhibit similar initial performance, shelf storage depends strongly on precursor solution stoichiometry. While all devices exhibit some degree of healing, bromide‐deficient films show a remarkable improvement, more than doubling in their photoconversion efficiency. Photoluminescence spectroscopy experiments performed under different atmospheres suggest that this increase is due, in part, to a trap‐healing mechanism that occurs upon exposure to the environment. The results highlight the importance of understanding and manipulating defects in lead halide perovskites to produce long‐lasting, stable devices.

## Graphical abstract

Herein, the effects of variations in precursor stoichiometry on the properties, performance, and stability of MAPbBr_3_ photovoltaic devices are examined. It is found that while the initial device performance is similar, devices with under‐stoichiometric composition are significantly improved upon storage in air without illumination, which is associated with defect healing.

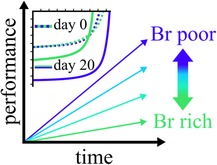

## 1 Introduction

Hybrid organic–inorganic lead halide perovskites have earned a lot of research attention in the past decade due to their broad‐spectrum absorption and efficient photocurrent generation in solar cells, with record performances reaching those of silicon (24.2% photoconversion efficiency, PCE, at the time of writing).^1^ In order to reach commercial potential, however, perovskite solar cells must achieve these high values reliably from device to device and, furthermore, must retain their performance at least long enough to recoup production costs. Despite predictions of high defect tolerance,^2^, ^3^ these two aspects remain elusive, with many factors playing overlapping roles in device behavior over time. It is known, for example, that the interactions at the device interfaces can contribute to device breakdown.^4^, ^5^, ^6^, ^7^ Another key aspect determining device stability is related to the microstructure of the perovskite active layer,^8^ with large, uniform grains having been shown to be more stable than small grains upon exposure to both oxygen^9^ and humidity.^10^ The composition of the active layer also plays an important role, with multication compositions showing an overall higher stability.^11^, ^12^, ^13^ Even when taking these factors into account, literature reports concerning device stability vary greatly even for devices fabricated from the same recipe in the same device architecture.^14^ This suggests that stability and reproducibility issues share a link, in which variation in the reproducibility of device performance may also lead to variation in its stability. For example, certain recipes for the fabrication of perovskite layers may result in nonhomogenous films, which may also serve to increase sample‐to‐sample variation.^15^ Recently, our group uncovered one key factor that adversely impacts the reproducibility and stability of MAPbI_3_ perovskite solar cells: the exact stoichiometric ratio of the precursor solution. We showed that purposefully adjusting the ratio between the precursor components (methylammonium iodide (MAI) and lead acetate trihydrate (Pb(Ac)_2_)) in small, almost negligible amounts results in large variations in the subsequent photovoltaic (PV) performance and stability.^16^ These stoichiometric variations are correlated with the photoluminescence (PL) behavior of the MAPbI_3_ thin films, displaying significant differences in both initial PL quantum efficiency (PLQE) and its evolution after exposure to light and oxygen.^17^

To date, much of the work on stability and performance has focused on the high achievers of the perovskite PV family: MAPbI_3_ and the triple‐cation film composition. While solar cells using methylammonium lead tribromide (MAPbBr_3_) perovskites show much lower PCE, its wide bandgap and corresponding high *V*_OC_ offer the potential for inclusion into tandem cells, where a MAPbBr_3_ absorber is combined with a narrow‐bandgap material to collect additional photons and achieve higher performance.^18^, ^19^, ^20^ Furthermore, its light emission at 545 nm is ideal for application in light‐emitting diodes (LEDs), as green is one of the fundamental pixel colors.^21^, ^22^, ^23^, ^24^, ^25^ Despite the critical importance of reproducibility and stability of MAPbBr_3_ films and devices, few reports exist addressing these issues. Apart from early works that suggest MAPbBr_3_ to be stable upon exposure to light and elevated temperature,^26^, ^27^ no systematic studies addressing the stability and reproducibility of such films could be found.

In this report, we carefully examine the effect of precursor solution stoichiometry on the properties, performance, and storage stability of MAPbBr_3_ thin films and devices. By deliberately and incrementally adjusting the ratio of MABr to Pb(Ac)_2_ in the precursor solution, similar to our previous work on MAPbI_3_, we tune the composition of the film from slightly bromide deficient to bromide excessive and examine the response of the active layer in PVs and LEDs. While the initial performance of devices shows little dependence on the stoichiometry of the precursor solution, the evolution of their performance upon storage varies drastically. Remarkably, the slightly understoichiometric bromide‐based films show a large degree of defect healing that is evident in both PLQE and device performance, resulting in a significant increase in its PCE upon storage. Our results underline the strong role that film composition plays in defining the properties, performance, and stability of devices and further promote the idea that defect engineering may be a viable strategy to produce desirable properties in perovskites.

## 2 Results and Discussion

### 2.1 Optoelectronic Properties

**Figure 1:**
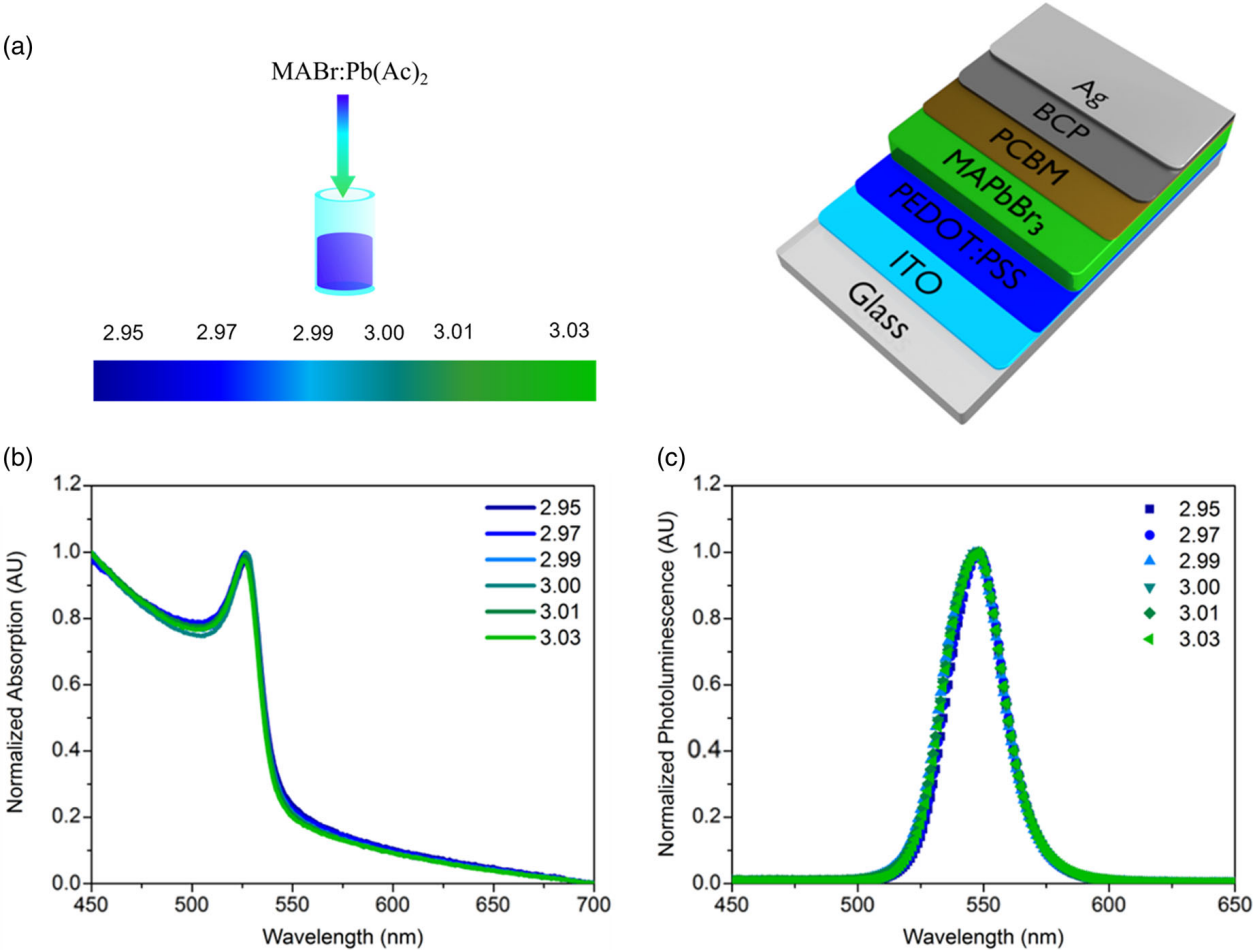
a) Schematic representation of stoichiometry variation and photovoltaic device structure; b) UV‐Vis absorption spectra; and c) photoluminescence spectra of MAPbBr_3_ films with stoichiometries of 2.95, 2.97, 2.99, 3.00, 3.01, and 3.03.

To tune the composition of MAPbBr_3_ films, we employed the same strategy as previously used for MAPbI_3_,^16^, ^17^ based on the one‐step lead acetate method for film fabrication, as depicted schematically in Figure 1a.^28^ This recipe has been shown to produce compact films with a highly uniform composition,^15^, ^29^ reducing pixel‐to‐pixel or spot‐to‐spot variation in measurements.^30^ Here, lead acetate and methylammonium bromide precursors are weighed and dissolved in DMF such that the molar ratio of MABr:Pb(Ac)_2_ (denoted as *y*) is under the “ideal” stoichiometry of 3, i.e., *y *= 2.95. Following the fabrication of the devices at *y *= 2.95, a small amount of MABr stock solution in DMF is added to the precursor solution such that the stoichiometry is now 2.97 MABr:Pb(Ac)_2_. By repeating this method, we create a series of films constituting the profile shown in Figure 1a: *y = *2.95, 2.97, 2.99, 3.0, 3.01, and 3.03 MABr:Pb(Ac)_2_. This range was selected as it represents an error in precursor solution stoichiometry of below 2%, making it small enough to be possibly introduced unintentionally during device fabrication. A more detailed description of this precursor preparation procedure is included in the supplementary information. This progression of solutions was used to create two types of thin films: one being glass/ITO/PEDOT:PSS/MAPbBr_3_ for the fabrication of solar cells and LEDs (a complete device in the inverted architecture with the top extraction layer and electrode shown in Figure 1a) and the other being glass/MAPbBr_3_, used for optical measurements. Together, these allow for a variety of measurements to understand the impact of precursor solution stoichiometry on thin‐film composition and properties in MAPbBr_3_ films.

One possible change that might be observed is the broadening or narrowing of the optical gap, which would be indicative of changes in the electronic structure of perovskite. As shown in Figure 1b, the UV‐Vis absorption of each film, the absorption onset at 550 nm and peak position near 530 nm are independent of solution stoichiometry; therefore, the bandgap is tolerant to the range of errors in the precursor composition introduced by varying *y*. The normalized photoluminescence spectra (Figure 1c) agree with this observation, demonstrating the same peak position and shape for each film. This is also the case for MAPbI_3_, where both under‐ and overstoichiometric films show an absorption onset of about 780 nm.^16^

**Figure 2:**
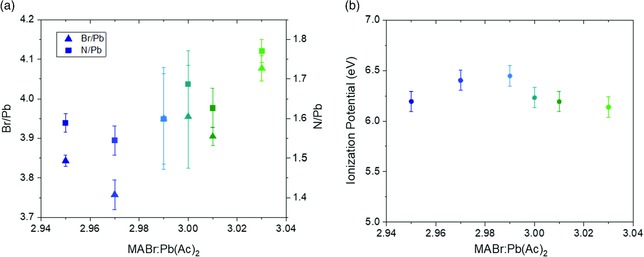
a) Br/Pb (triangles) and N/Pb (squares) ratios measured by X‐ray photoemission spectroscopy and b) ionization potential measured by UV photoemission spectroscopy for MAPbBr_3_ films with different stoichiometries.

The impact of the precursor solution stoichiometry on film surface composition is, however, directly observed by X‐ray photoelectron spectroscopy (XPS), as shown in Figure 2a. Here, the intensity of Pb4f_7/2_, Br3d_5/2_, and N1s peaks was tracked for seven spots on two films of each *y*, allowing us to qualitatively assess surface uniformity in addition to surface chemistry (with the full survey being shown in Figure S1, Supporting Information). As shown by the triangles in Figure 2a, the ratio between atomic percentages of Br:Pb increases slightly with increasing MABr:Pb(Ac)_2_ in solution, ranging from 3.75 to a maximum of 4.1. Similarly, the amount of methylammonium at the surface, deduced by tracking the ratio of N to Pb, also increases, ranging from 1.55 to a maximum of 1.75 for the overstoichiometric films. These trends agree with those observed for MAPbI_3_; however, I:Pb and N:Pb ratios in these films increase more rapidly, rising at a rate of 0.075 per 0.01 change in *y*, whereas bromide films only rise at a rate of 0.05 per 0.01 change in *y*. Comparable error bars indicate that the surface of the films is uniform both across a single sample and between multiple samples. Notably, the ionization potential (Figure 2b) is largely unchanged over the range of stoichiometries presented here. This is in contrast with the results obtained for MAPbI_3_ films, where the ionization potential increases monotonically by 0.3 eV over the range Δ*y* = 0.12.^16^

### 2.2 Microstructure

**Figure 3:**
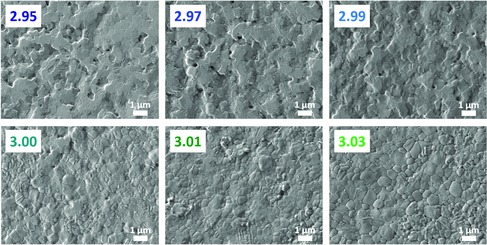
SEM images of MAPbBr_3_ films with different stoichiometries.

The microstructure of films often corresponds to the properties observed; for example, a high *J*_SC_ often correlates with a large grain size.^31^ Therefore, we evaluated the surface structure of the films via scanning electron microscopy, shown in Figure 3. Unlike its iodide counterparts, where the microstructure is largely unchanged over Δ*y* = 0.1,^16^, ^17^ the microstructure of MAPbBr_3_ films (where Δ*y* = 0.08) changes heavily as a function of solution stoichiometry. Understoichiometric (*y* = 2.95–2.99) films are smooth at the surface and lacking in well‐defined grains, but as *y* increases, grains start to appear, ranging from nanometer scale to 1 μm in size. The films for all *y* exhibit complete substrate coverage, lacking in pinholes.

### 2.3 Photovoltaic Performance and Stability

**Figure 4:**
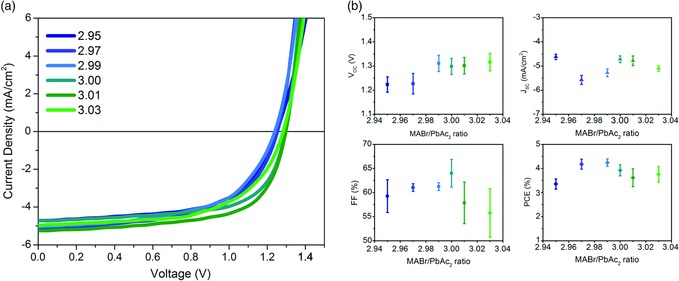
a) J–V characteristics of representative photovoltaic devices with the structure glass/ITO/PEDOT:PSS/MAPbBr_3_/PCBM/BCP/Ag. The stoichiometry of the MAPbBr_3_ active layer varied between 2.95 and 3.03. b) Statistics of the photovoltaic performance of the devices.

Despite changes in film microstructure and chemical composition at the surface, the initial performance of photovoltaic cells was highly tolerant to changes in *y* (Figure 4, with device structure shown in Figure 1a). The open‐circuit voltage (*V*_oc_; Figure 4b) of understoichiometric films is somewhat lower than that of overstoichiometric films; however, this difference approaches sample‐to‐sample variation. This observation coincides with the lack of change observed in the ionization potential (Figure 2b) and bandgap (Figure 1b,c). The short‐circuit current density (*J*_sc_), fill factor (FF), and PCE are all approximately constant as *y* varied. These results contrast with the MAPbI_3_ films, where an increase in the MAI:Pb(Ac)_2_ ratio (and the I:Pb ratio on the surface of the films) coincides with an increase in the *V*_OC_, varying linearly over a range of 0.2 V for Δ*y* = 0.1, with the PCE following suit.^16^

**Figure 5:**
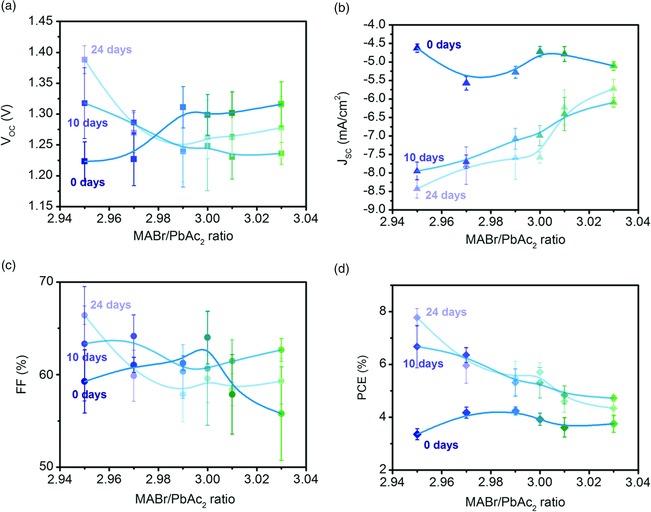
Evolution of the photovoltaic performance after 10 and 24 days of shelf storage for devices with different stoichiometries: a) open‐circuit voltage; b) short‐circuit current density; c) fill factor; and d) power conversion efficiency.

Following the initial measurements, the devices were covered and placed on a shelf in the lab, to be remeasured again at 10‐ and 24‐day intervals. Interestingly, they display stark differences due to *y* when aged over the course of several weeks. As shown in Figure 5a, the change in *V*_OC_ over time depends on the precise film composition, with *y* = 2.95 films continually increasing over the time period measured. The *V*_OC_ for other *y* diminishes over time or diminishes and then heals slightly, with no observable trend. The fill factor remains roughly constant within the pixel‐to‐pixel variation. The most apparent trend appears when examining *J*_sc_. For higher *y*, the current improves slightly, by about 1 mAcm^−2^. As *y* decreases, such that the precursor solution and films contain less Br, *J*_sc_ improvement is much more drastic. The most understoichiometric film, *y* = 2.95, has an initial value of −4.5 mAcm^−2^; at 24 days, this value nearly doubled at −8.5 mAcm^−2^. These strong changes result in the clear trend observed in the PCE, where the initial performance for all *y* is between 3.5% and 4%, but at 10 and 24 days, the *y* = 2.95 films have more than doubled, showing a PCE of almost 8%. The highest stoichiometric films (*y* = 3.03) only increase from 4% to 5% PCE over this time period, with the change in PCE versus *y* displaying a linear increase with decreasing Br content. For a longer shelf life of over 100 days, the films retain this property of highly increased performance for low *y* and slightly increased performance for high *y*, suggesting the possibility for long‐lived devices when combined with proper encapsulation strategies. This shelf storage behavior is very different for MAPbI_3_ films, where *V*_oc_ increases between 10 and 20 days for all but the highest stoichiometry studied (*y* = 3.075), and *J*_sc_ and PCE both decrease over time for all *y* (i.e., no such healing is observed).^16^

**Figure 6:**
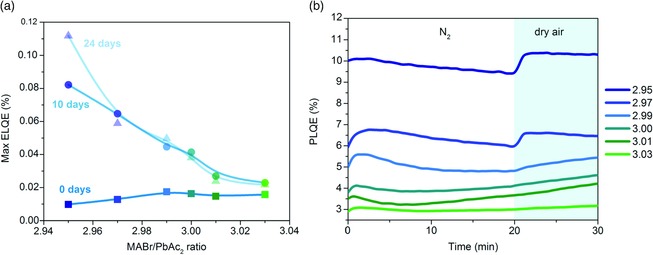
a) Evolution of maximum measured ELQE of the photovoltaic devices presented in Figure 5. b) Evolution of the PLQE of MAPbBr_3_ films during exposure to N_2_ (first 20 min) followed by exposure to dry air under continuous illumination.

When operated as LEDs, MAPbBr_3_ devices show similar trends in their maximum electroluminescent quantum efficiency (ELQE) (Figure 6a). Initial measurements are constant at 0.01%, but after 24 days, the *y* = 2.95 films improve to 0.11%, while the *y* = 3.03 films only improve slightly, to 0.02%. To gain some insight into what mechanisms might be causing this healing, we measured PLQE under a 405‐nm continuous excitation with a power density of ≈80 mWcm^−2^ over time and under different atmospheres. As shown in Figure 6b, understoichiometric films display the highest PLQE, with *y* = 2.95 at 10%. PLQE decreases with increasing *y*, with *y* = 3.03 exhibiting 3% PLQE. These values are consistent with observations of iodine films, where understoichiometric films exhibited higher PLQE than overstoichiometric films.^16^ Such a behavior is likely a result of increased formation probability of deep trap states for halide‐rich films,^2^, ^32^ increasing the rate of nonradiative recombination. For bromide films, PLQE remains constant through 20 min of continuous measurement under nitrogen flushing. When the atmosphere is switched to dry air, understoichiometric films show rapid improvement, while the overstoichiometric films show only very small increases in PLQE or no change at all. We note that for all stoichiometries, we observe no shift in the PL peak position or change in its spectral shape throughout the experiment (Figure S2, Supporting Information). This suggests that the changes in PLQE are associated with defect healing, rather than changes in the emission properties of perovskite layers.

This PLQE healing behavior is also seen for MAPbI_3_ films under exposure to oxygen atmosphere.^16^, ^17^ A possible explanation has been attributed to the diffusion of oxygen into iodine vacancy sites and the subsequent formation of a superoxide species.^33^, ^34^ This species is noted to be the appropriate size to fill the vacancy and is thought to be responsible for an initial boost in PLQE under oxygen exposure (and later decomposition of the film into lead halide, the organic cation, water, and oxygen). A similar mechanism could be responsible for the increase in PLQE in MAPbBr_3_ films, with the smaller overall healing as compared to I‐based films resulting from the shorter B—Pb bond length, subsequent lattice stabilization, and lower prevalence of defect states deep in the bandgap.^2^, ^3^, ^35^, ^36^

When comparing the effect of solution stoichiometry on the properties of MAPbBr_3_ perovskites and those of MAPbI_3_, two key differences stand out. First, the microstructure of the former is highly sensitive to even minute changes in stoichiometry, while in the case of MAPbI_3_ films, nearly no variations in grain size or structure were observed. This should assist researchers in identifying MAPbBr_3_ films made from solutions of slightly different stoichiometries and facilitate in increasing their reproducibility. The second stark difference is in the storage stability of devices, and since in both cases the solar cell architecture and extraction layers were kept the same, the differences are likely to originate from different active layer microstructures and densities of defect states. The microstructure of the perovskite active layer has been shown to influence its stability with degradation processes commencing at grain boundaries in both O_2_ 9 and humid environments.^10^ In the case of MAPbI_3_, films of all stoichiometries exhibited the same microstructure, and so it is predominantly the different densities of ionic defects that determined the storage stability of these devices. In the case of MAPbBr_3_, both the microstructure and the density of defects vary, making assignments more complex. However, it is interesting to note that understoichiometric MAPbBr_3_, which did not exhibit clear grain structure, is also the most stable, in agreement with the previously observed initiation of degradation at the grain boundaries. This suggests that for these samples, defect healing results in increased photovoltaic performance, while the smooth grain boundary‐free microstructure delays the onset of degradation. Given the high degree of overlapping phenomena between the microstructure, PV performance, PLQE, and their behavior over time, further research is needed to clearly elucidate the mechanisms at play in this healing behavior of the films. What our experiment clearly shows is that understoichiometric films display a high degree of improvement over time, indicating that the manipulation of defect states within the bulk film could be an effective strategy for increased film and device lifetime.

## 3 Conclusion

In conclusion, we examined the properties, performance, and shelf stability of MAPbBr_3_ films and devices as functions of changing precursor solution stoichiometry. While the initial PV performance is constant over changing *y*, films with a small bromide deficiency undergo a large increase in performance, with their PCE more than doubling in value from 3.5% to nearly 8%. Films with excess bromine also improve with prolonged shelf storage, but only by 1%. The maximum ELQE shows a similar trend. PLQE measurements under nitrogen and dry air indicate that there is likely a trap healing mechanism at play, though the details of such a process remain to be closely examined. Though the overall trends are different than those of the MAPbI_3_ films, both sets of results indicate the strong role that compositional variation plays in device longevity and film properties, suggesting that purposeful integration of defects into perovskite films may promote their eventual use.

## 4 Experimental Section

4.1

4.1.1

##### Sample and device fabrication

MAPbBr_3_ precursor solutions were prepared following the method introduced in previous works.^16^, ^17^ In short, MABr was mixed with lead acetate trihydrate (PbAc_2_•3H_2_O) at an understoichiometric ratio, and stoichiometry was changed by adding specific amounts of MABr to the solution. A detailed explanation of this procedure can be found in Supplementary Note 1. Photovoltaic devices were fabricated using prepatterned glass/ITO substrates (PsiOTech Ltd.) that were cleaned by ultrasonication in acetone and 2‐propanol for 10 min each. The substrates were then blow dried and O_2_ plasma cleaned (100 W, 0.4 mbar) for 10 min. PEDOT:PSS (Heraeus) was spin coated on the substrates at 4000 rpm for 45 s and annealed on a hot plate at 150 °C for 10 min in the ambient condition. The samples were then transferred into a dry‐air glovebox in which MAPbBr_3_ active layers were deposited by spin coating at 2000 rpm for 60 s. Immediately after spin coating, the samples were blow dried with a dry air gun for 30 s and left to dry on an aluminum holder at room temperature for 5 min. Afterward, the samples were annealed for 5 min at 80 °C. Next, the samples were transferred into a N_2_‐filled glovebox, in which PCBM (20 mg mL^−1^ in chlorobenzene) electron transporting was spin coated dynamically at 2000 rpm for 45 s, followed by a 10 min annealing at 80 °C. A BCP (0.5 mg mL^−1^ in 2‐propanol) hole‐blocking layer was spin coated at 4000 rpm for 25 s. The devices were completed by thermal evaporation of an 80 nm‐thick Ag electrode. Samples for spectroscopic and microscopic measurements were fabricated in an identical fashion to the active layer of the PV devices but using glass substrates for UV‐Vis and PL and glass/ITO/PEDOT:PSS for SEM, XPS, and UPS.

##### Device characterization

The devices were characterized as solar cells under simulated AM 1.5 sunlight at 100 mW cm^−2^ irradiance (Abet Sun 3000 Class AAA solar simulator) with a Keithley 2450 source measure unit. Light intensity was calibrated with a Si reference cell (NIST traceable, VLSI) and corrected by measuring the spectral mismatch between the solar spectrum, spectral response of the perovskite solar cell, and the reference cell. The mismatch factor was calculated to be around 10%. When characterized as LEDs, the devices were measured inside an integrating sphere (Labsphere Inc.). The current–voltage characteristics were measured using a source measure unit (Keithley 2450). At the same time, the emitted light spectra were recorded using a scientific‐grade spectrometer (Ocean OpticsQE65Pro). The optical system (integrating sphere, spectrometer, and coupling optical fiber) was calibrated using a calibrated light source (Ocean Optics HL‐2000‐CAL).

##### Photoemission spectroscopy

MAPbBr_3_ samples were transferred into an ultrahigh vacuum chamber of the PES system (ThermoScientific ESCALAB 250Xi) for measurements. The samples were exposed to air only for a short time span of ≈30 s. All measurements were performed in the dark, and several spots on each sample were measured to ensure enough statistics. Ultraviolet photoelectron spectroscopy measurements were carried out using a double‐differentially pumped He discharge lamp (*hv* = 21.22 eV) with a pass energy of 2 eV and a bias at −10 V. XPS measurements were performed using an XR6 monochromated AlKα source (*hv* = 1486.6 eV) and a pass energy of 20 eV.

##### UV‐Vis spectroscopy

Optical absorption spectra were measured with a Jasco UV‐660 spectrophotometer in the range from 400 to 700 nm. Absorption of the substrate was subtracted as a baseline correction.

##### Photoluminescence spectroscopy

PLQE measurements were carried out inside an integrating sphere (LabSphere) with excitation by a 405 nm CW laser (Coherent). The spectra were recorded using a QE65 Pro (Ocean Optics) spectrometer.

##### Scanning electron microscopy

SEM was performed using a JSM‐7610F FEG‐SEM (Jeol). Samples were mounted on standard SEM holders using conductive Ag paste to avoid sample charging. Images were recorded using a secondary electron detector (LEI) at an acceleration voltage of 1.5 kV and a chamber pressure <10^−6^ mbar.

## Conflict of Interest

The authors declare no conflict of interest.

## Supporting information

Supplementary
