# Supplementary material for: Effect of Precursor Stoichiometry on the Performance and Stability of MAPbBr3 Photovoltaic Devices
Source: Energy Technol (Weinh). 2019 Aug 20;8(4):1900737. doi: 10.1002/ente.201900737 (PMC7188293; doi:10.1002/ente.201900737)
Supplement: Supplementary file 1 — Supplementary [file ENTE-8-1900737-s001.docx]

Copyright WILEY-VCH Verlag GmbH & Co. KGaA, 69469 Weinheim, Germany, 2018.

Supporting Information

Effect of Precursor Stoichiometry on the Performance and Stability of MAPbBr_3_ Photovoltaic Devices

Lukas M. Falk, Katelyn P. Goetz, Vincent Lami, Qingzhi An, Paul Fassl, Jonas Herkel, Fabian Thome, Alexander D. Taylor, Fabian Paulus and Yana Vaynzof*

1. **Solution preparation procedure**

The changes in stoichiometry were performed by adding small volumes of a MABr/DMF stock solution into precisely known volumes of MABr:Pb(Ac)_2_/DMF perovskite precursor solution, as previously shown^[1,2]^. The adapted procedure is described in detail below.

In order to perform controlled stoichiometric changes, it is necessary to know the total solid concentration in each of the solutions. Therefore, the density of the two solutions was determined in the following fashion: for the MABr/DMF stock solution, 314.6 mg MABr was dissolved in 1 ml DMF (25 wt%). The density of this solution was determined (by weighing exactly known volumes) to be 1114.6 mg/ml, i.e. a solid concentration of 278.65 mg/ml MABr. For the precursor solution, carefully weighed amounts of MABr and Pb(Ac)_2_ were dissolved in DMF at 42 wt% with an initial stoichiometry of 2.95:1. After the addition of HPA (6.43 μl / 1 ml DMF) with a density of 1.206 mg/ml, the weight percentage of perovskite (MABr+PbAc2) decreases to 41.8%. The density of the various stoichiometries was again determined by weighing known volumes (shown in Table S1). Once the density of the precursor solution and the perovskite weight percentage are known, the solid concentration as well as the amount of Pb(Ac)_2_ per substrate (40 µl precursor solution per) can be calculated for each stoichiometry y. Appropriate amounts of the MABr/DMF stock solution were then added to the precursor solution in order to achieve the desired changes in stoichiometry y (see Table S1 for an example calculation).

This is by far the most reproducible and accurate method to perform controlled stoichiometric changes, and furthermore it allows the use of the same precursor solution for a complete batch, limiting the effect of human error in preparing solutions. As the initial precursor weighing, and the pipetting using calibrated Gilson Pipetman Microman E pipettes (for determining the density as well as performing changes in stoichiometry), both introduce small errors on the order of ~1%, we were able to estimate the systematic error for each stoichiometry y. As an example, using the values in Table S1, even in the extreme case that the actual volume per sample was 38 µl instead of the supposed 40 µl (5% relative error), the final stoichiometry after 5 variation steps would still be y = 3.0579 instead of y = 3.06, which represents an error of only .069%.

| **Step** | **Mass PbAc2 (mg)** | **Density (mg/mL)** | **Perovskite Content (wt %)** | **Pb(Ac)2 per 1 mL** | **Pb/40 μl (mg)** | **MABr mass needed for next step (mg)** | **Vol. stock MABr needed for next step (μl)** |
| --- | --- | --- | --- | --- | --- | --- | --- |
| 2.95 (x2) | 272.70 | 1229.25 | 41.83 | 274.87 | 10.99 | 1.48 | 5.31 |
| 2.97 (x2) | 250.71 | 1228.59 | 41.74 | 273.25 | 10.93 | 1.35 | 4.85 |
| 2.99 (x2) | 228.85 | 1227.93 | 41.66 | 271.68 | 10.87 | 0.61 | 2.19 |
| 3.00 (x2) | 207.11 | 1227.60 | 41.61 | 270.90 | 10.84 | 0.55 | 1.96 |
| 3.01 (x2) | 185.44 | 1227.28 | 41.57 | 270.12 | 10.80 | 0.97 | 3.47 |
| 3.03 (x2) | 163.82 | 1226.64 | 41.48 | 268.59 | 10.74 | X | X |

**Table S1**: Example calculation to perform the change in stoichiometry for the MAPbBr_3_ samples used in the paper. The 2.95 stoichiometry solution contains 272.70 mg PbAc2 and 237.5 mg MABr, dissolved in 746.3 µl DMF with 4.04 µl HPA. Two samples using 40 µl volume are prepared at each step; after the second sample, the volume of stock MABr/DMF solution in the right column is added to reach the next stoichiometry.

1. **XPS Measurement, Survey Spectra**

**
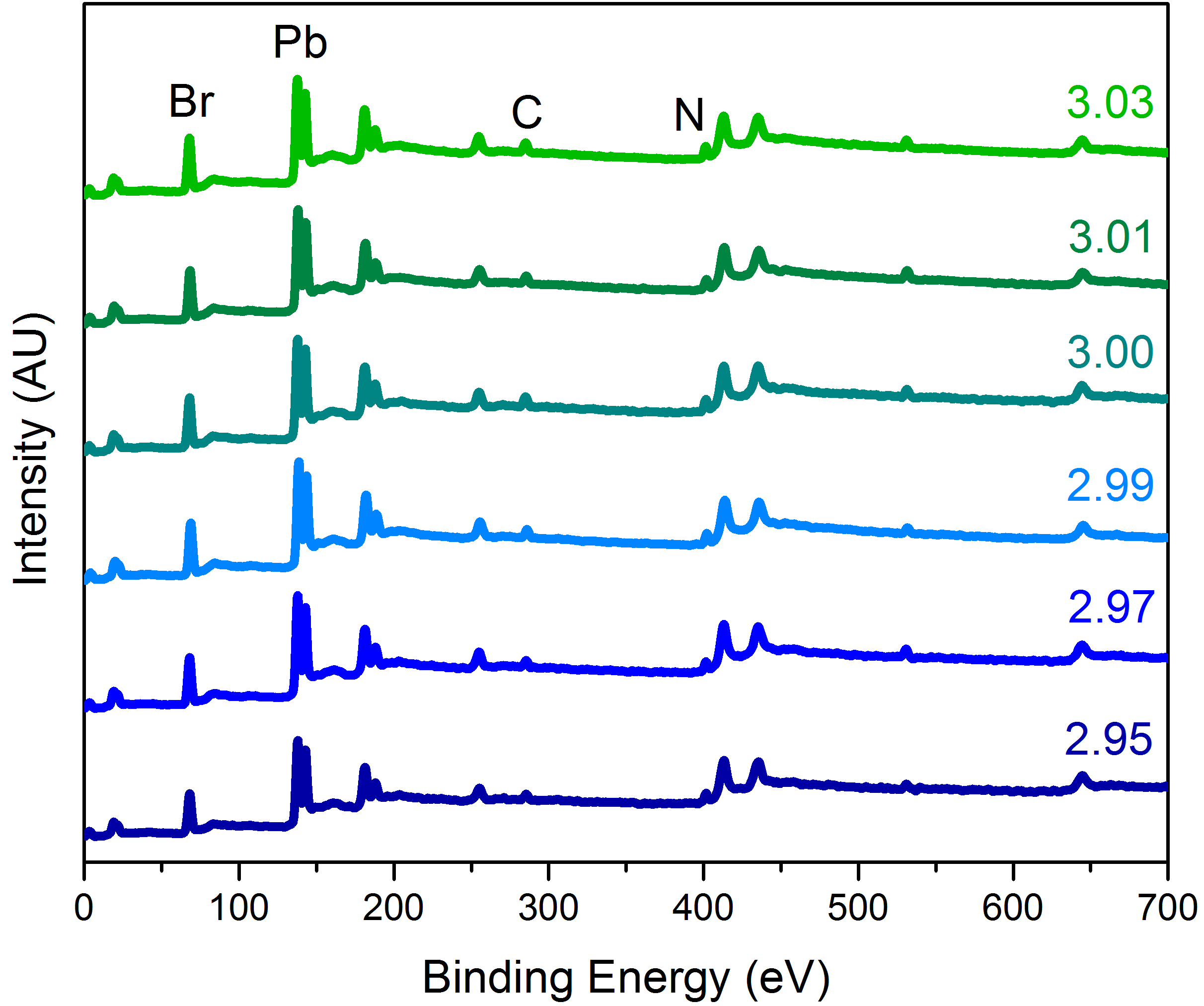
**

**Figure S1.** The X-ray photoelectron spectroscopy survey for all films measured, with the Br, Pb, C, and N characteristic binding energies labeled.

1. **PL spectra**


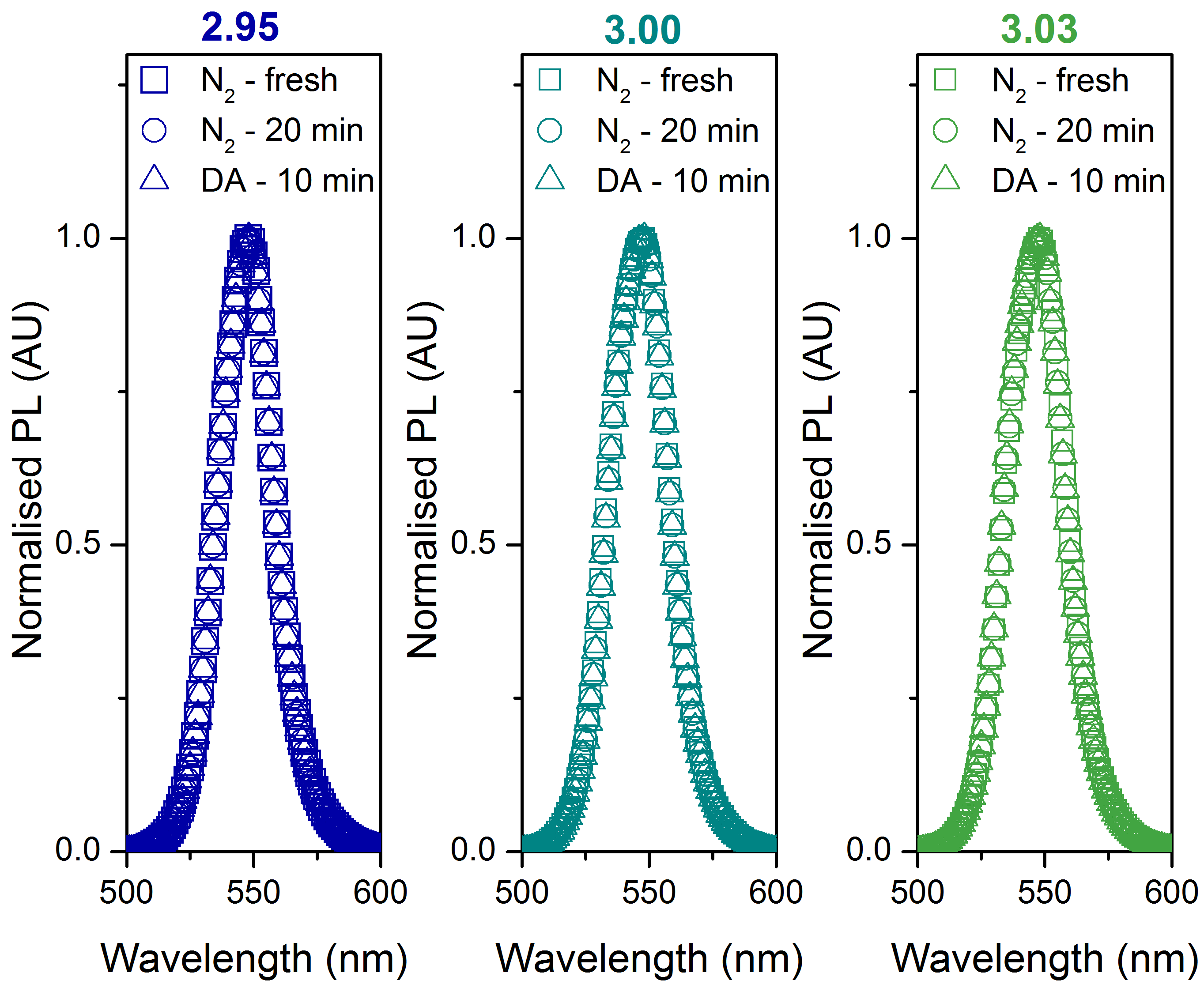


**Figure S2:** Normalised PL spectra for samples of stoichiometry 2.95, 3.00 and 3.03 as measured in N2 at time 0, in N2 after 20 min and after additional 10 min in dry air. No shifts or changes to the spectral shape are observed.

**References**

[1] P. Fassl, V. Lami, A. Bausch, Z. Wang, M. T. Klug, H. J. Snaith, Y. Vaynzof, *Energy Environ. Sci.* **2018**, *11*, 3380.

[2] P. Fassl, Y. Zakharko, L. M. Falk, K. P. Goetz, F. Paulus, A. D. Taylor, J. Zaumseil, Y. Vaynzof, *J. Mater. Chem. C* **2019**, *7*, 5285.
